# Endoglin-Mediated Suppression of Prostate Cancer Invasion Is Regulated by Activin and Bone Morphogenetic Protein Type II Receptors

**DOI:** 10.1371/journal.pone.0072407

**Published:** 2013-08-13

**Authors:** Michael J. Breen, Diarmuid M. Moran, Wenzhe Liu, Xiaoke Huang, Calvin P. H. Vary, Raymond C. Bergan

**Affiliations:** 1 Department of Medicine, Northwestern University, Chicago, Illinois, United States of America; 2 Robert H. Lurie Cancer Center, Northwestern University, Chicago, Illinois, United States of America; 3 Center for Molecular Innovation and Drug Discovery, Northwestern University, Chicago, Illinois, United States of America; 4 Center for Molecular Medicine, Maine Medical Center Research Institute, Scarborough, Maine, United States of America; University of Kentucky College of Medicine, United States of America

## Abstract

Mortality from prostate cancer (PCa) is due to the formation of metastatic disease. Understanding how that process is regulated is therefore critical. We previously demonstrated that endoglin, a type III transforming growth factor β (TGFβ) superfamily receptor, suppresses human PCa cell invasion and metastasis. Endoglin-mediated suppression of invasion was also shown by us to be dependent upon the type I TGFβ receptor, activin receptor-like kinase 2 (ALK2), and the downstream effector, Smad1. In this study we demonstrate for the first time that two type II TGFβ receptors are required for endoglin-mediated suppression of invasion: activin A receptor type IIA (ActRIIA) and bone morphogenetic protein receptor type II (BMPRII). Downstream signaling through these receptors is predominantly mediated by Smad1. ActRIIA stimulates Smad1 activation in a kinase-dependent manner, and this is required for suppression of invasion. In contrast BMPRII regulates Smad1 in a biphasic manner, promoting Smad1 signaling through its kinase domain but suppressing it through its cytoplasmic tail. BMPRII’s Smad1-regulatory effects are dependent upon its expression level. Further, its ability to suppress invasion is independent of either kinase function or tail domain. We demonstrate that ActRIIA and BMPRII physically interact, and that each also interacts with endoglin. The current findings demonstrate that both BMPRII and ActRIIA are necessary for endoglin-mediated suppression of human PCa cell invasion, that they have differential effects on Smad1 signaling, that they make separate contributions to regulation of invasion, and that they functionally and physically interact.

## Introduction

Prostate cancer (PCa) is the most common cancer and the second leading cause of cancer mortality for US males [1], with essentially all deaths resulting from metastatic disease [2]. Metastasis is a highly inefficient process in which cells must overcome numerous barriers, an initial one of which is escape from the site of origin through the acquisition of an invasive phenotype [3]. Signaling through the TGFβ superfamily and its associated receptors is a key regulator of this process in numerous cancer types [4], including PCa [5].

TGFβ is the prototypical member of a family of extracellular ligands – of which there are 33 in mammals – that regulate numerous developmental and homeostatic processes, and do so through a relatively conserved signaling mechanism [6]. With canonical TGFβ signaling, ligand binding induces oligomerization of dimers of serine/threonine kinase type I and type II receptors (RIs and RIIs, respectively), wherein constitutively active RIIs then phosphorylate RIs. These activated RIs then phosphorylate downstream receptor-associated Smads (R-Smads). The phosphorylated R-Smad subsequently binds to the common mediator, Smad4, and the resultant complexes affect gene transcription. Broadly speaking, the signaling through this superfamily can be subdivided into TGFβ-like ligands whose cognate RIs tend to be activin receptor-like kinase (ALK)4, 5, or 7, tending to activate R-Smads Smad2 or 3, and bone morphogenetic protein (BMP)-like ligands signaling through cognate RIs ALK1, 2, 3, or 6, and R-Smads Smad1, 5, or 8.

Endoglin (also referred to as CD105) is a homodimeric transmembrane protein that acts as an auxiliary TGFβ superfamily receptor, and is considered a type III receptor [7]–[9]. Endoglin modulates signaling downstream of TGFβ and BMP ligands, tending to promote signaling preferentially through BMP-like pathways, while inhibiting TGFβ-like pathways [10]–[14]. In addition, endoglin regulates numerous cellular processes through non-Smad dependent pathways. Pertinent to cellular invasion, endoglin interacts through its cytoplasmic domain with the LIM-domain-containing proteins zyxin and zyxin-related protein 1 to regulate focal adhesion composition and the actin cytoskeleton [15], [16]. Moreover, endoglin regulates integrin activation and signaling in a number of cellular processes [17]–[21]. Endoglin can also modulate the transforming potential of H-Ras [22].

The role of endoglin in cancer is complex, given differential expression and function across cell types [23], [24]. Most studies of endoglin function have been conducted in endothelial cells. Germline mutations in endoglin cause the genetic disease hereditary hemorrhagic telangiectasia [25], highlighting endoglin’s role as a key regulator of endothelial cell motility, invasion, and proliferation. In multiple cancers including PCa, endoglin is overexpressed in endothelial cells of the microvasculature and is associated with angiogenesis [26]–[28]. It is also highly expressed in the stromal microenvironment [29]. Thus, at the overall tissue level, endoglin is often overexpressed in cancer. In contrast and of high importance, in epithelial cells of multiple solid tumors – and in prostate epithelium in particular – we and others have demonstrated that endoglin expression is lost with disease progression [30], [31]. We have shown that this loss promotes cell detachment [31], cell invasion [14], [32] and metastasis [33]. Mechanistically, we have shown that endoglin suppresses invasion in a manner that is dependent upon the RI ALK2 and the downstream R-Smad Smad1 [14]. However, the RIIs involved in this process remain unknown.

Given the role of ALK2 and Smad1 in endoglin-mediated suppression of invasion (EMSI) in PCa, we hypothesized that one or more RIIs are involved in this process. In this report we identify that ActRIIA and BMPRII are required. Interestingly, they have opposite effects on downstream Smad1 signaling. ActRIIA promotes the previously-identified signaling axis, while BMPRII has bimodal function, promoting Smad1-dependent signaling via its kinase activity while inhibiting it via its large cytosolic tail domain. Together these findings are the first to identify ActRIIA and BMPRII as important regulators of EMSI and demonstrate that they operate through distinct yet interdependent mechanisms. These results open new avenues for pharmacologic targeting of PCa metastatic potential.

## Materials and Methods

### Cell Culture & Transfection

The origin and culture conditions for PC3-M human PCa cells have been described [34]. The PC3-M line is a highly metastatic PC3-derivative cell line. They were maintained in RPMI 1640 media supplemented with 2 mM L-glutamine, 10 mM HEPES buffer, 50 units/ml penicillin, 50 µg/ml streptomycin, and 10% fetal bovine serum (Life Technologies, Grand Island NY). DU145 cells are human PCa cells derived from a brain metastasis and were obtained from ATCC (Manassas, VA). These cells were maintained in DMEM media supplemented with the above mentioned products in addition to 1 mM sodium pyruvate (Life Technologies). All cells were maintained at 37°C in a humidified atmosphere of 5% carbon dioxide and 95% air under sub-confluent exponential growth conditions with triweekly changes of medium, and were replaced with fixed-passage cells on a regular basis.

Cell lines were authenticated according to methods described in the American Type Culture Collection Technical Bulletin No. 8, Cell Line Verification Test Recommendations [35]. Specifically, cells from low passage (i.e.,<15 passages) frozen stocks were used and were replenished after 20 passages; cells underwent routine microscopic examination to confirm uniform and standard cellular architecture and no microbial infection; and cells were tested (within three months) and found negative for mycoplasma infection.

Transient transfection of cells was performed as previously described [36]. Briefly, 24 hours after plating, cells were transfected with TransIT-LT1 Transfection Reagent (Mirus Bio LLC, Madison, WI) for invasion assays involving plasmid DNA only, with Dharmafect Duo (Thermo Scientific, Lafayette, CO) for invasion assays involving simultaneous delivery of plasmid DNA and siRNA, or with Dharmafect2 (Thermo Scientific, formerly Dharmacon) for invasion and luciferase experiments involving delivery of siRNA alone. For immunoprecipitation experiments, cells were transfected with plasmid DNA using Lipofectamine LTX (Life Technologies). Cells were then used in the indicated assays 24–48 hours after transfection. All reagents were used according to manufacturers’ instructions. In some experiments, as indicated, cells were washed twice with PBS, serum-starved in media containing 0.1% bovine serum albumin for 3 hours, and treated with 2 ng/ml TGFβ, 5 ng/ml BMP7, or 20 ng/ml BMP9 for 30 minutes prior to lysis.

### Reagents

Neutralizing antibody to ActRIIA, Fc-ActRIIA and Fc-BMPRII ligand traps, and recombinant human TGFβ were purchased from R&D Systems (Minneapolis MN). Antibodies to the following proteins were purchased: phospho-Smad1/5, phospho-Smad1/5/8, Smad1, and Myc-tag from Cell Signaling Technology (Danvers, MA), endoglin from BD Biosciences (San Jose, CA), GAPDH from Enzo Life Sciences (Farmingdale NY), FLAG-tag and Myc-tag from Sigma-Aldrich (St. Louis, MO), α-tubulin from Santa Cruz Biotechnology (Santa Cruz, CA), HRP-conjugated goat anti-mouse IgG (H+L) F(ab')2 fragment from KPL (Gaithersburg, MD), and ECL donkey anti-rabbit whole antibody from GE Healthcare Biosciences (Pittsburgh, PA). The siRNAs used in this study were all pools of four individual siRNAs, ordered from Thermo Scientific as ON-TARGETplus SMARTpools.

### Plasmids

The following expression vectors were used: pCDNA3 empty vector purchased from Life Technologies, endoglin in a pCDNA3 vector, was constructed and previously described by us [31], pCMV-β-galactosidase (β-gal) purchased from Agilent Technologies (Santa Clara, CA), pCDNA3-ActRII, pCDNA3-ActRIIA-ΔKD-5myc, pCDNA3-ActRIIB and pCDNA3-ActRIIB-ΔKD-5myc were generously provided by Wylie Vale (Salk Institute, La Jolla, CA) [37], [38], pCDNA3-ActRIIA-myc was generated from pCDNA3-ActRIIA by Custom DNA Constructs (University Heights, OH), pCMV5-BMPRII, -BMPRII-KI and –BMPRII-Δtail constructs were generously provided by Liliana Attisano (University of Toronoto, Canada) [39], pGL3-MLP-BRE_2_-luciferase was a generous gift from Peter ten Dijke (Leiden University Medical Centre, Netherlands) [40], pRL-TK-*Renilla* luciferase was purchased from Promega (Madison, WI).

### Invasion Assays

Invasion assays were performed essentially as described previously by us [41], with the following modifications. Briefly, cells were co-transfected with the indicated DNA and β-gal, with or without siRNA as indicated. After 48 hours, cells were plated onto 8.0 µm pore Growth Factor-Reduced Matrigel Invasion Chambers (BD Biosciences) in serum-free media containing 0.1% BSA, in replicates of N = 4 wells for each experimental condition. Serum-free NIH-3T3 conditioned media was placed in the bottom chamber as a chemoattractant, and cells were allowed to invade for 24 hours. Cells on top of the membrane were removed from three wells per condition (allowing quantification of invaded cells) while the fourth was left undisturbed (total cell controls). After fixing cells and staining for β-gal expression using an In Situ ß-Galactosidase Staining Kit (Agilent Technologies), nine microscopic fields (of 100x) per well were imaged on an Olympus CKX41 microscope equipped with a QImaging RETIGA 1300 digital CCD camera and QCapture imaging software. β-gal positive cells were counted in each field using Image J software and normalized invasion was determined for each well as βgal^+^ cells in invasion well/β-gal^+^ cells in total well. Relative invasion was calculated as a fraction of a control condition (e.g. empty vector/siNeg).

### Quantitative Reverse Transcriptase Polymerase Chain Reaction

RNA was isolated from cells using RNeasy Mini Kit (QIAGEN, Valencia, CA), per manufacturer’s instructions. RNA was treated with RNase free DNase and its quality and quantity assessed by optical density. cDNA was synthesized using either TaqMan Reverse Transcription Reagents (Life Technologies) or qScript cDNA SuperMix (Quanta Biosciences, Gaithersburg, MD), and qPCR performed using TaqMan Universal PCR Master Mix and TaqMan primer-probe pairs on a 7500 Real Time PCR System (all from Life Technologies), all as previously described by us [33]. RT minus control reactions were run as a negative control. Validated gene specific exon spanning primer/probe sets for ACVR2A, ACVR2B, BMPRII, TGFβRII, endoglin, Smad1, Smad5, Smad8, and GAPDH were from Applied Biosystems. Assays were run in replicates of 2 and the resultant mean threshold cycles (Ct) were used for further analysis. Assays were repeated at least once at a separate time, also in replicates of 2. The Ct for individual reactions was identified through Applied Biosystems 7500 Real Time PCR System software. Gene expression was normalized to that of GAPDH, and relative gene expression was calculated by the 2^–ΔΔCt^ method [42].

### Luciferase Assays

Cells were transiently transfected with pGL3 BRE_2_-luciferase (inducible) and pRL-TK (constitutive) plasmids at a ratio of 20∶1, with additional plasmids ± siRNAs as indicated, as previously described by us [14]. Briefly, after 48 hours cells were lysed and luciferase activity was measured using a Dual-Luciferase Reporter Assay System (Promega). Measurements were made on either a Synergy HT plate reader (BioTek, Winooski, VT) or a Monolight 2010 luminometer (Analytical Luminescence Laboratory, San Diega CA). After subtracting background signal, luciferase activity was calculated as ratio of firefly luciferase/*Renilla* luciferase.

### Western Blotting

Cell lysis and immunoblotting were performed as previously described by us [14]. Briefly, cells were washed with PBS, lysed with lysis buffer: PBS (137 mM NaCl, 10 mM Na phosphate, 2.7 mM KCl), 0.5% Triton X-100, 1 mM EDTA, 2.5 mM sodium pyrophosphate, 1 mM β-glycerophosphate with addition of protease inhibitor cocktail (#P8340,) phosphatase inhibitor cocktails 1 and 2, 10 mM sodium fluoride, and 1 mM sodium orthovanadate (all from Sigma-Aldrich), and lysates clarified by centrifugation, all at 4°C. Protein concentration was determined by the Bradford method (Bio-Rad, Hercules, CA), equal amounts were separated by SDS-PAGE under denaturing and reducing conditions, transferred to nitrocellulose (Bio-Rad), and stained with Ponceau S (Sigma-Aldrich) to verify even loading and transfer. Membranes were either blocked and probed with primary (4°C overnight) and secondary antibody (1hr room temperature) in 5% milk in TBS-T (25 mM Tris-HCl, pH 7.4, 150 mM NaCl, 0.1% Tween 20) or blocked and probed with 0.5% milk/TBS-T using SNAP i.d. vacuum manifold (Millipore), per manufacturer’s suggestions. Membranes were then incubated with ECL Western Blotting Detection Reagents and exposed to Amersham Hyperfilm ECL (both from GE Healthcare). Films were developed using a SRX-101A film processor (Konica Minolta, Wayne, NJ). Membranes were stripped in 62.5 mM Tris (pH 6.8), 2% SDS, and 100 mM β-mercaptoethanol for 20 minutes at 50°C, washed briefly in TBS-T, and re-probed as above. All Western blots were repeated at least once, at a separate time.

### Immunoprecipitation

Cells were washed with PBS and surface proteins were crosslinked with 2 mM water-permeable crosslinking reagent 3,3′-Dithiobis(sulfosuccinimidylpropionate) (DTSSP; Thermo Scientific) in PBS. Crosslinking reaction was quenched by addition of Tris, pH 7.5 to 20 mM. Cells were then lysed as above using buffer composed of the following: 10 mM Tris HCl, pH 7.4; 145 mM NaCl; 10% glycerol; 0.5% NP-40; protease and phosphatase inhibitors as above. Lysates were cleared by centrifugation and protein concentration was determined by the Bradford method as above. An equal amount of protein was precleared with agarose beads conjugated to recombinant Protein A (Life Technologies; used for rabbit IgG) or Protein G Plus (Thermo Scientific; used for mouse IgG) for 1 hr at 4°C with rotation. Precleared lysates were transferred to new tubes and incubated with IgG overnight at 4° with rotation. Antibody-antigen complexes were recovered by 2 hr incubation with Protein A or Protein G, in concordance with the preclearance step. Beads were recovered and supernatant was removed and saved. Beads were washed three times with ice-cold lysis buffer. Samples were equilibrated to 1X Laemmli buffer containing 5% β-mercaptoethanol (Sigma-Aldrich) and incubated in 95°C heat block for 6 min, reducing and denaturing the samples and cleaving the crosslinker. Samples were Western blotted as described above.

### Statistical Analysis

To analyze invasion assays one-tailed unpaired Student’s t-tests were calculated based on the assessed phenotype of reversing suppression of invasion. For luciferase assays and qRT-PCR, Student’s two-tailed unpaired Student’s t-tests were used. P values≤0.05 were considered significant.

## Results

### Endoglin-Mediated Suppression of Invasion Requires ActRIIA and BMPRII

**Figure 1:**
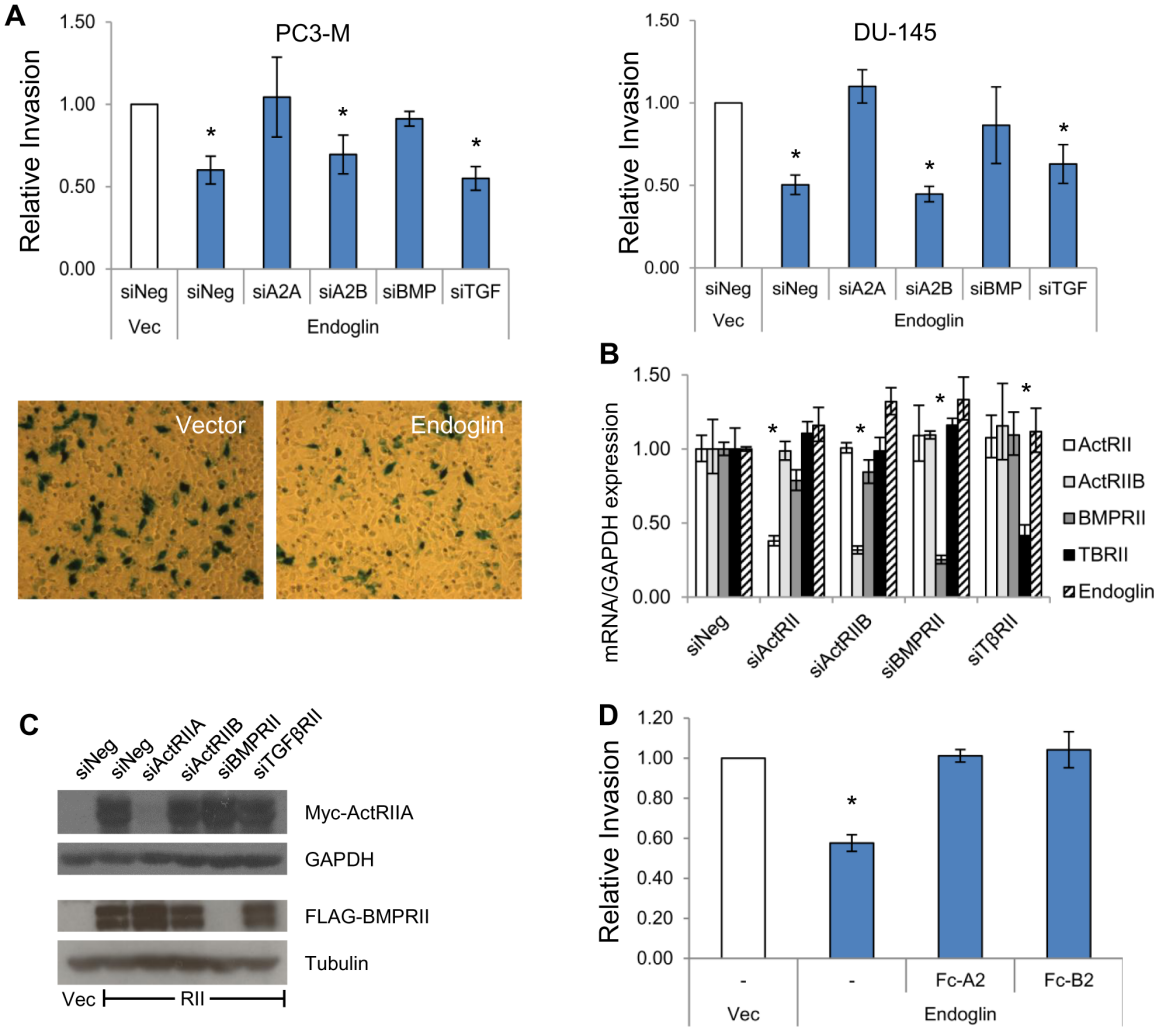
Endoglin requires ActRIIA and BMPRII to suppress invasion. **A)** The effect of type II receptors on endoglin mediated suppression of invasion (EMSI). PC3-M (left) or DU-145 (right) cells were transiently transfected with empty vector (Vec) or with endoglin along with siRNA, as indicated. The following siRNAs were used: siNeg – non-targeting negative control, si2A - targets ActRIIA, si2B - targets ActRIIB, siBMP - targets BMPRII, siTGF - targets TGFβRII. After 48 hours, cell invasion was measured. Data represent the mean ± SEM of 3 independent experiments, each in replicates of 3. *, p≤0.05 compared to Vec/siNeg. Micrographs are representative images of cells that have invaded through Matrigel, were stained for βgal, and imaged (magnification 100X). **B**) ActRIIA and BMPRII siRNA is specific. PC3-M cells were transiently transfected with endoglin along with the indicated siRNAs as in (A). After 48 hours mRNA expression was assessed via qRT-PCR, normalized to GAPDH, and expressed relative to siNeg-transfected cells (normalized to 1.0). Data represent the mean ± SD of a single experiment, performed in replicates of N = 2; similar results were seen in an independent experiment (N = 2 replicates). *, p≤0.05 compared to siNeg. **C**) ActRIIA and BMPRII siRNA suppresses target protein. PC3-M cells were transfected with ActRIIA-myc (upper panels) or BMPRII-flag (lower panels), as well as with the indicated siRNAs, followed by Western blot for indicated proteins. Data are from a representative experiment (N = 2 separate experiments). **D**) Blocking ActRIIA or BMPRII ligand binding inhibits EMSI. PC3-M cells were transiently transfected with empty vector or endoglin as above. After 2 days, cells were pretreated for 5 hrs ligand traps comprised of ActRIIA or BMPRII extracellular domain fused to immunoglobulin Fc region (Fc-A2 or Fc-B2 respectively). Treatment continued during the subsequent conduct of cell invasion assays. Data represent mean ± SEM of 3 independent experiments, each in replicates of N = 3. *, p≤0.05 compared to Vec/-.

Endoglin-mediated suppression of invasion (EMSI) in PCa requires a RI, i.e., ALK2 [14]. In addition, canonical signaling through the TGFβ superfamily of receptors requires ligand-dependent activation of the RI by a RII [6]. We therefore hypothesized that EMSI would require one or more RIIs. We evaluated this by transfecting PC3-M and DU-145 human PCa cells with endoglin, along with siRNA specific to individual RII subtypes: activin A receptor type IIA (ActRIIA), activin A receptor type IIB (ActRIIB), bone morphogenetic protein receptor type II (BMPRII), or transforming growth factor β receptor type II (TGFβRII). For both PC3-M and DU-145 cells, endoglin significantly suppresses invasion to 60% and 50% of control cells, respectively, and this is abrogated by siRNA targeting ActRIIA or BMPRII but not ActRIIB or TGFβRII (Figure 1A). In order to further investigate the mechanism of ActRIIA and BMPRII in affecting EMSI, studies focused upon human PC3-M PCa cells. They constitute a metastatic phenotype [34] and are known to express very low baseline levels of endoglin [14].

There is close homology amongst TGFβ superfamily receptors. It is therefore particularly important to address siRNA specificity, which we demonstrate in Figure 1B. Using sequence specific primers for each receptor, we show by qRT-PCR that receptor specific siRNA significantly knocks down the targeted receptor by ≥60% in each instance, with no significant modulation of non-target receptors. The efficacy and specificity of siRNA targeting ActRIIA and BMPRII was next demonstrated by measuring effects at the protein level. An endemic problem within the field relates to the fact that antibodies raised against a given TGFβ superfamily receptor subtype tend to have relatively high levels of cross reactivity. We addressed this by co-transfecting cells with either myc-tagged ActRIIA or FLAG-tagged BMPRII, along with siRNA to individual RIIs, followed by tag-specific Western blot (Figure 1C). In this manner we demonstrate receptor-specific siRNA-mediated suppression of protein expression to nearly undetectable levels for both ActRIIA and BMPRII.

Binding of extracellular ligands to ActRIIA and BMPRII constitutes a main determinant of their signaling function. We therefore hypothesized that if ActRIIA and BMPRII are truly important regulators of EMSI, then their modulation of this process should be dependent upon cognate ligands, at least in part. We determined that this is in fact the case by transfecting cells with endoglin, followed by measuring the effect on invasion when extracellular cognate ligands were blocked from receptor binding (Figure 1D). This was accomplished by treating cells with recombinant protein constructs, Fc-A2 or Fc-B2, consisting of the ActRIIA or BMPRII extracellular domains, respectively, fused to an immunoglobulin constant domain, thus serving as a ligand trap. As can be seen in Figure 1D, blocking of ligand binding to either receptor reverses EMSI. These ligand-blocking studies complement our knockdown studies. Taken together, our findings implicate ActRIIA and BMPRII as important physiologic regulators of EMSI.

### ActRIIA and BMPRII Have Opposite Effects on Downstream Smad1 Signaling

**Figure 2:**
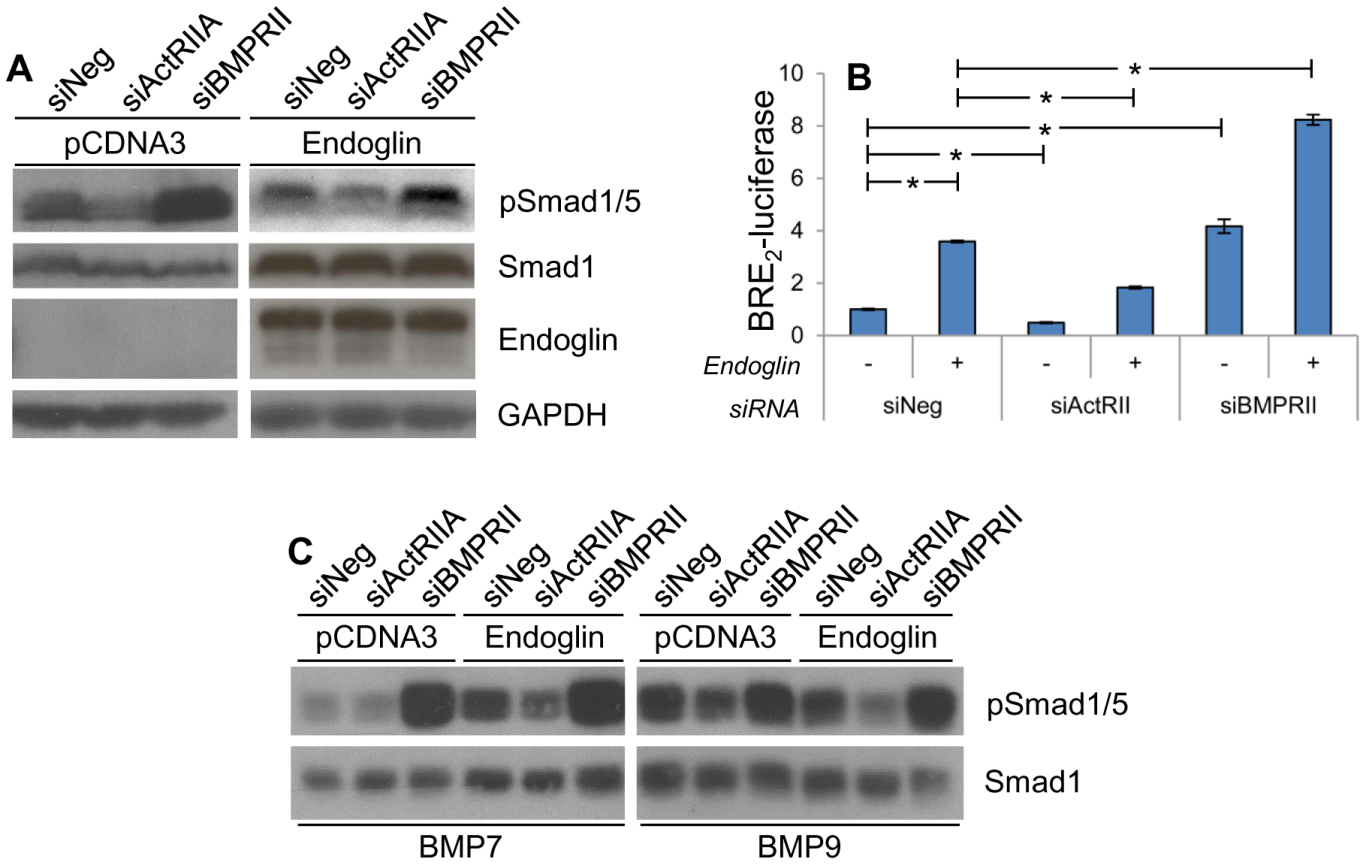
ActRIIA promotes Smad1 signaling while BMPRII is inhibitory. PC3-M cells were transiently transfected with empty vector or endoglin and the indicated siRNA as in Figure 1. Two days later cells were lysed for Western blot (**A**) or luciferase promoter assay (**B**). **A**) ActRIIA and BMPRII differentially regulate Smad1 protein phosphorylation. Western blot on resultant cell lysate was performed for Smad1, phospho-Smad1/5 (pSmad1/5), endoglin and GAPDH. Data are from a representative experiment (N = 4 experiments). **B**) ActRIIA and BMPRII differentially regulate BRE_2_-luciferase activation. Cells were additionally co-transfected with BRE_2_-luciferase and *Renilla* luciferase constructs, and luciferase activity (normalized to *Renilla* luciferase activity) was measured. Data are the mean ± SD from a single experiment conducted in replicates of N = 2, conducted three separate times with similar results (also N = 2). *, p≤0.05 between the indicated groups. **C**) BMP7- and BMP9-stimulated Smad1 phosphorylation is differentially regulated by ActRII and BMPRII. Cells were transfected as above, serum-starved, and treated with BMP7 or BMP9 as indicated. Western blot on resultant cell lysate was performed for phospho-Smad1/5 (pSmad1/5) and total Smad1. Data are from a representative experiment (N = 2 experiments).

We have previously demonstrated that endoglin increases phosphorylation of Smad1, that Smad1 suppresses cell invasion, and that Smad1 is necessary for EMSI [14]. We therefore evaluated the effect of ActRIIA and BMPRII on the regulation of Smad1 phosphorylation. This was done by knocking down ActRIIA or BMPRII via transfection of PC3-M cells with siRNA while co-transfecting with empty vector or endoglin. Phosphorylation of Smad1 was then assessed by Western blot (Figure 2A). Irrespective of endoglin status, knockdown of ActRIIA decreases phospho-Smad1 levels. Surprisingly, knockdown of BMPRII has the opposite effect; it increases phospho-Smad1. As with our previous studies [31], the endogenous endoglin expression in PC3-M cells is so low as to approach the limit of detection.

We have previously demonstrated in the same system we are currently using that primary induced increases in endoglin expression status induce increases in both Smad1 phosphorylation as well as in Smad1 transcriptional activity, as measured by luciferase reporter assay using the Smad1-responsive BRE_2_-luciferase reporter construct [14]. Importantly, in this same system, we have also demonstrated how several different perturbations have discordant effects upon Smad1 phosphorylation and its functional transcriptional activity. However, in all instances, changes in Smad1 transcriptional function reflected concordant effects upon biological function, as evaluated by associated Smad1 knockdown studies as well as invasion assays [14], [32]. While the mechanism underlying this phenomenon is not entirely clear, it appears to reflect the fact that human prostate cells contain very high levels of acid phosphatase, and that during cell lysis it has protein-specific effects that cannot be adequately brought in check even with high levels of phosphatase inhibitors [43]. We therefore consider assessment of Smad1 transcriptional function to be the informative assay. As such, we went on to evaluate the effect of ActRIIA and BMPRII knockdown on BRE_2_-luciferase activation (Figure 2B). Again we show that, irrespective of endoglin status, knockdown of ActRIIA significantly decreases Smad1 function while knockdown of BMPRII significantly increases it. These findings are consistent with the Smad1 phosphorylation data in Figure 2A, and demonstrate that changes in Smad1 phosphorylation are associated with altered Smad-mediated transcription. However, they also demonstrate that BMPRII-induced effects upon Smad1 transcriptional function are not congruent with BMPRII’s effect upon EMSI.

To investigate BMPRII further, we examined effects upon signaling under conditions of bone morphogenic protein (BMP) ligand stimulation. ActRIIA and BMPRII were knocked down as in Figure 2A, cells were serum-starved, simulated with either BMP7 or BMP9, and Smad1/5 phosphorylation assessed by Western blot (Figure 2C). With either BMP7 or BMP9, knockdown of ActRIIA attenuates ligand-stimulated Smad1/5 phosphorylation, while knockdown of BMPRII augments it. By demonstrating a similar signaling response profile under conditions of BMP ligand stimulation compared to that of standard culture conditions, the importance of BMP is further supported. These findings corroborate those in Figure 1D, which demonstrate that BMP ligand is necessary for EMSI.

**Figure 3:**
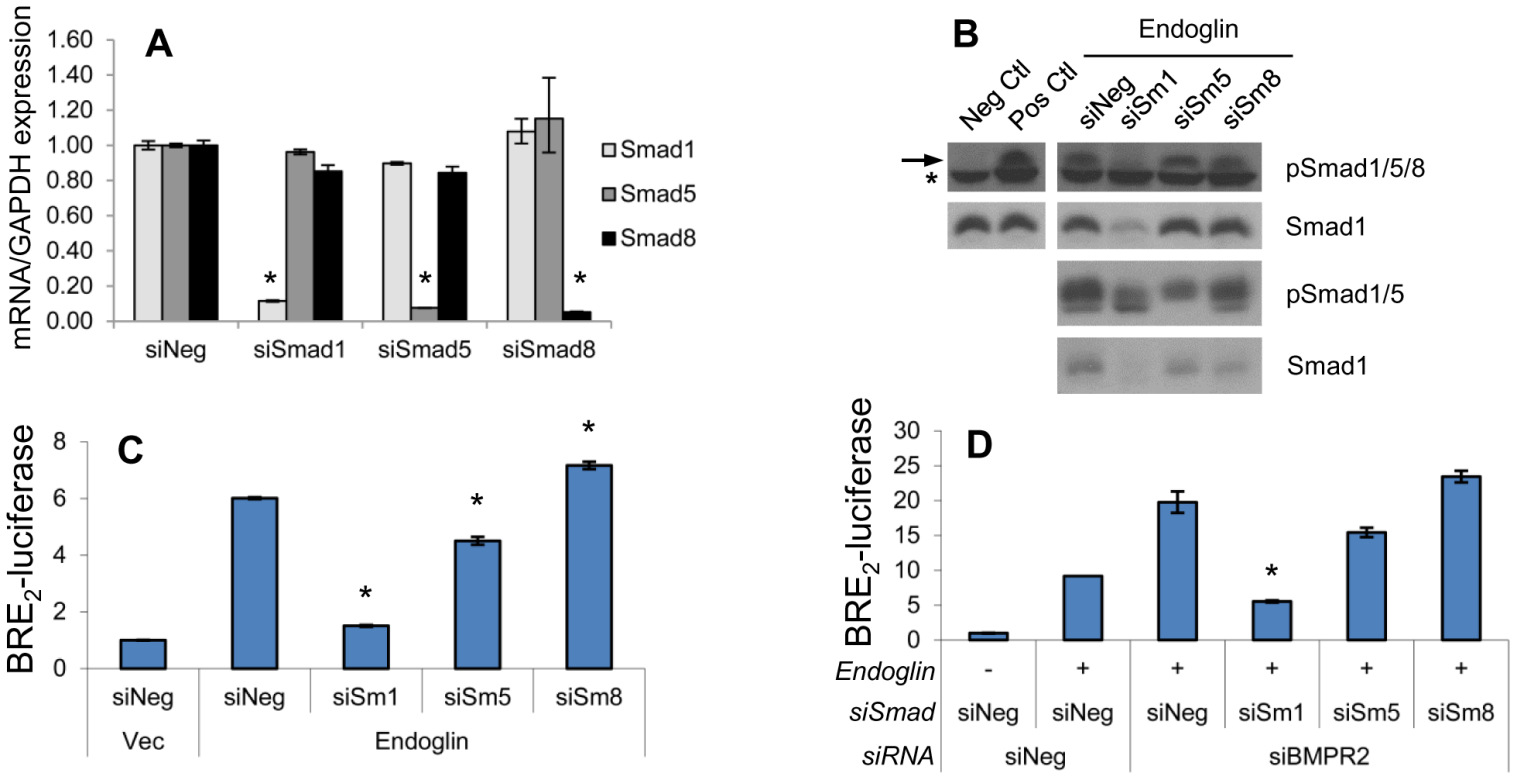
Smad1 is the main downstream target of endoglin. PC3-M cells were transfected with endoglin, vector (Vec), or with siRNA to Smad1, (siSm1), Smad5 (siSm5), Smad8 (siSm8), BMPRII (siRII) or non-targeting (siNeg), and processed 48 hrs later as indicated. **A**) Smad-targeting siRNA suppresses transcript in a Smad-specific fashion. Smad1, -5, and -8 mRNA expression was assessed via qRT-PCR, normalized to GAPDH, and expressed relative to siNeg-transfected cells (normalized to 1.0). Data represent mean ± SD from a single experiment conducted in replicates of N = 2, that was repeated 3 separate times (also in replicates of N = 2) with similar results. *, p≤0.05 compared to siNeg. **B**) Effect of siRNA on phospho-Smad1/5/8, phospho-Smad1/5, and total Smad1 protein levels. Cell lysates were probed by antibody directed towards phospho-Smad1/5/8 (pSmad1/5/8) and total Smad1 protein by Western blot. The non-specific band (*) immediately under the pSmad1/5/8 band (arrow) confirms even loading. Negative control cells (Neg Ctl) were transfected with vector and serum starved overnight. Positive control cells (Pos Ctl) were transfected with endoglin, not serum starved and were treated with TGFβ for 30 min. Separate samples were similarly transfected and treated, and cell lysates were probed for phospho-Smad1/5 (pSmad1/5). Data are from one representative experiment in each case, repeated 3 separate times with similar results. **C**) Endoglin-mediated BRE_2_-luciferase activity is largely mediated by Smad1. Cells were transfected with endoglin and were additionally co-transfected with BRE_2_- and *Renilla* luciferase construct, and luciferase assays performed. Data represent mean ± SD of a single representative experiment conducted in replicates of N = 2, repeated 3 separate times (replicates of N = 2) with similar results. *, p≤0.05 compared to Endoglin/siNeg. **D**) BMPRII-mediated suppression of BRE_2_-luciferase activity is largely mediated by Smad1. Cells were transfected as in (C) with addition of indicated siRNA and luciferase activity as assessed as above. Data represent mean ± SD of a single representative experiment conducted in replicates of N = 2, repeated 2 separate times (replicates of N = 2) with similar results. *, p≤0.05 compared to Endoglin/siNeg/siBMPRII.

Neither the BRE_2_-luciferase promoter system nor the currently available phospho-Smad antibodies are able to completely distinguish between Smad1, Smad5 and Smad8 isoforms. We therefore assessed the extent to which these other Smad isoforms could account for the currently observed effects. Specifically, given that BMPRII knockdown unexpectedly led to what appeared to be increased Smad1 signaling, we wanted to examine the possibility that BMPRII knockdown was increasing Smad5 or Smad8 signaling, potentially masking a functionally important decrease in Smad1 signaling. Using gene-specific qRT-PCR analysis, we first demonstrate that siRNA to the individual Smad isoforms is highly specific and efficacious, significantly silencing the targeted isoform by ≥90% in each instance, while having no significant effect on non-target isoforms (Figure 3A). We next sought to determine which Smads were activated upon endoglin overexpression. Knockdown of Smad1 results in near total loss of specific signal from an antibody that recognizes phospho-Smad1, 5, and 8 (Figure 3B). We verify that Smad1 protein expression is effectively suppressed by siRNA to Smad1, but is not suppressed by siRNA targeting Smad5 or Smad8. Using an antibody that recognizes both phosphorylated Smad1 and Smad5, we demonstrate that Smad5 is also phosphorylated in the presence of endoglin. We then went on to demonstrate that knockdown of Smad1 causes a 90% reduction of the endoglin-induced increase in BRE_2_-luciferase (Figure 3C). In contrast, endoglin-induced increase in BRE_2_-luciferase activity is diminished by only 30% following Smad5 knockdown, and not at all by Smad8 knockdown. Finally, as we show in Figure 2 that BMPRII knockdown increases Smad1 activation, we examined the effect of Smad isoform suppression in the face of BMPRII knockdown in endoglin replete cells (Figure 3D). Similar to findings in Figure 3C, Smad1 knockdown significantly abrogates the increased BRE_2_-luciferase activity achieved by BMPRII knockdown, while Smad5 or Smad8 knockdown have no significant effect. All together the above findings demonstrate that ActRIIA increases Smad1 phosphorylation and function, while BMPRII decreases it. Smad5 appears to participate in these signaling events, but its impact is small and of borderline significance in the current system.

### ActRIIA-mediated Promotion of Smad1 Signaling is Dependent Upon Its Kinase Domain While BMPRII-mediated Inhibition of Signaling is Dependent Upon Its Tail Domain

**Figure 4:**
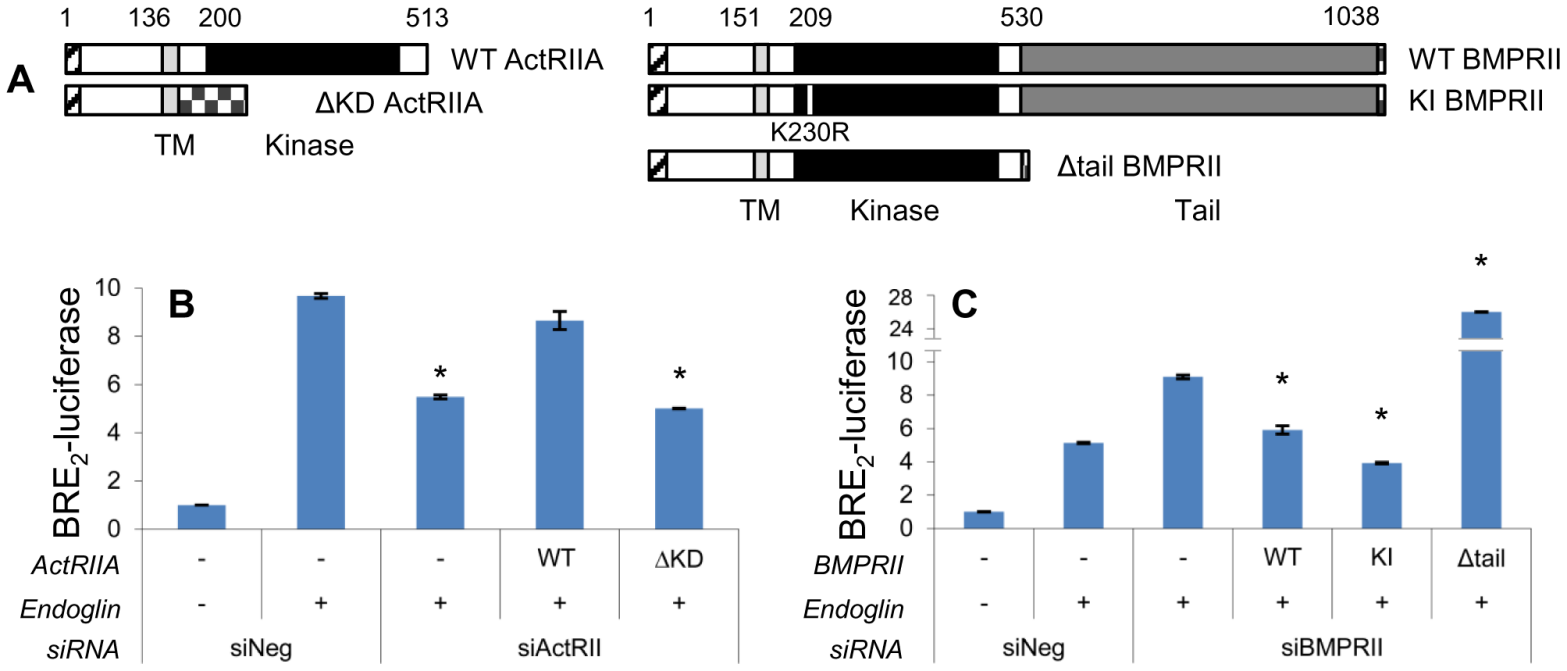
ActRIIA promotion of Smad1 signaling is kinase dependent, while BMPRII inhibition occurs via the tail domain. **A)** Schematic depiction of ActRIIA and BMPRII constructs. Signal peptide (hatched), transmembrane domain (light gray), kinase domain (black), and BMPRII tail domain (dark gray) are indicated with the amino acid position that begins each portion. Also indicated are five sequential Myc tags or single FLAG tag at the C-terminus of ΔKD ActRIIA and BMPRII constructs, respectively (checkered). The small white stripe in KI BMPRII’s kinase domain represents the site of kinase-inactivating mutation. Segment lengths are to scale. **B**) ActRIIA promotes Smad1 signaling dependent on the kinase domain. PC3-M cells were transfected with BRE_2_-luciferase and *Renilla* luciferase, endoglin, wild type (WT) or kinase domain deletion (ΔKD) ActRIIA constructs, and siRNA to ActRIIA or non-targeting as indicated. Luciferase assay performed as in Figure 2B. Data represent mean ± SD of a single representative experiment (N = 2 replicates), repeated twice (N = 2 replicates) with similar results. *, p≤0.05 compared to Eng/siNeg. **C**) BMPRII suppresses Smad1 signaling independent of kinase function but dependent on the tail domain. PC3-M cells were transfected as above except that BMPRII constructs and siRNA were used. WT  =  wild type; KI  =  kinase inactive; Δtail  =  tail domain deleted. Luciferase assay performed as in Figure 2B. Data represent mean ± SD of a single representative experiment (N = 2 replicates), repeated twice (N = 2 replicates) with similar results. *, p≤0.05 compared to Eng/siBMPRII.

A diagram of ActRIIA and BMPRII primary structure is depicted in Figure 4A. Both RIIs contain extracellular ligand-binding and intracellular kinase domains of comparable location and size. An important difference is that BMPRII contains a large tail domain of 508 amino acids that is not present on other RIIs.

We next conducted studies to control for potential siRNA off-target effects. Data presented below demonstrates that the level of receptor expression was crucial in determining function. Thus we adopted a strategy of simultaneously transfecting siRNA and plasmid to restore expression to near-endogenous levels. For each construct, we empirically determined the ideal concentration of the two reagents to achieve this goal (Figure S1). Unless otherwise stated, these conditions were used in all experiments in which exogenous receptor is used to replace silenced endogenous protein. Rescue experiments were performed in which wild type (WT) ActRIIA and BMPRII were re-expressed from plasmids in cells treated with siRNA targeting ActRIIA and BMPRII, respectively. As can be seen in Figures 4B and 4C, re-expression of WT ActRIIA or BMPRII reverses the effects on BRE_2_-luciferase activity of siRNA targeting ActRIIA or BMPRII, respectively. These findings demonstrate that the observed effects on Smad1 transcriptional activity are due to the loss of ActRIIA or BMPRII.

In order to better understand the mechanism by which RIIs mediate their effect upon Smad1 in the current system, we then went on to examine the role of specific RII domains. We assessed the ability of a series of mutant constructs, schematically depicted in Figure 4A, to restore function in the face of siRNA-mediated knockdown of endogenous ActRIIA or BMPRII. As shown in Figure 4B, siRNA targeting ActRIIA decreases endoglin-promoted BRE_2_-luciferase activity, and this is restored by re-expressing WT ActRIIA. However, ActRIIA lacking the kinase domain (ΔKD-ActRIIA) loses all such efficacy. These results indicate that the ability of ActRIIA to promote the endoglin-mediated increase in Smad1 transcriptional activity is dependent upon its kinase domain. Interestingly, we observed contrasting results when examining the importance of BMPRII domains. Both WT and kinase-inactive (KI) BMPRII revert the effect of BMPRII-siRNA on BRE_2_-luciferase activity (Figure 4C). However, deletion of the BMPRII tail domain (Δtail) not only leads to loss of efficacy, but in fact augments the effect of BMPRII-siRNA. These findings demonstrate that Smad1 signaling is regulated by ActRIIA in a manner dependent upon kinase activity and by BMPRII in a manner independent of kinase activity but dependent upon the tail domain.

### BMPRII Signals in a Bimodal Fashion

**Figure 5:**
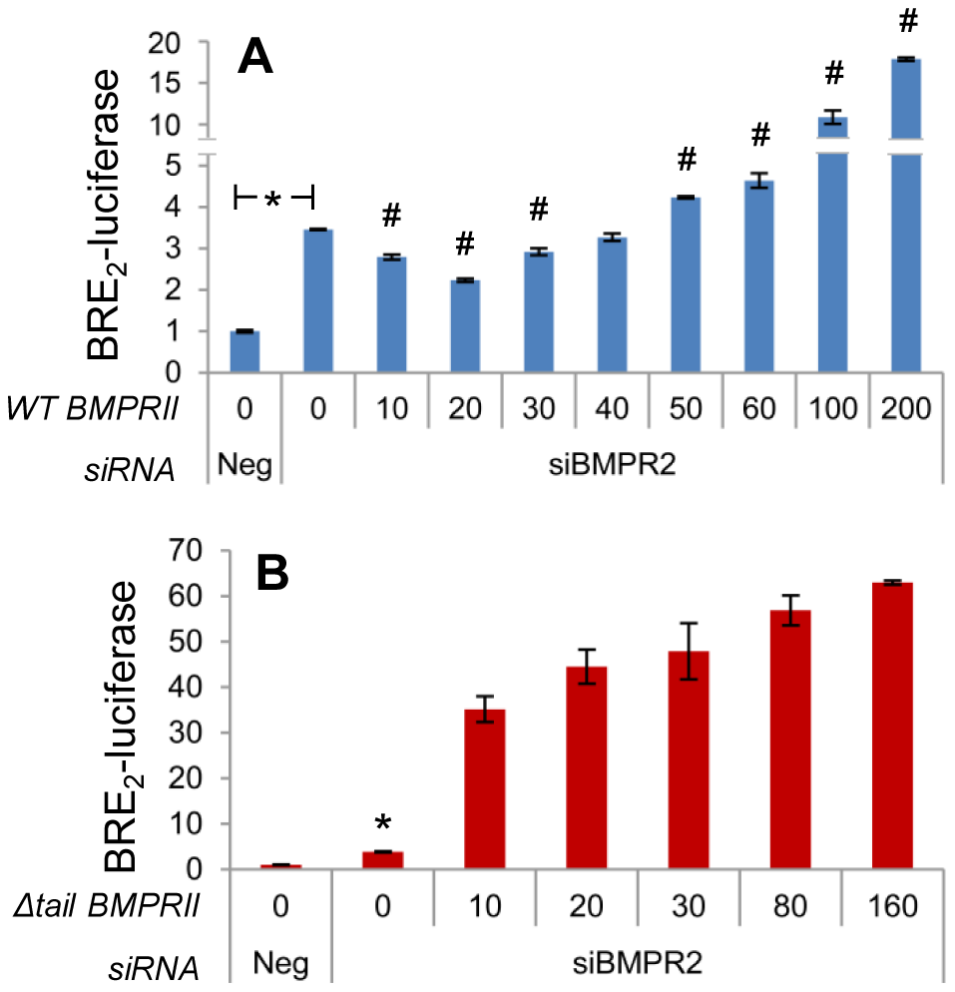
BMPRII suppresses Smad1 signaling in a dose- and tail domain-dependent manner. **A)** BMPRII suppresses BRE_2_-luciferase activity in a dose-dependent manner. PC3-M cells were transfected with indicated siRNA and BRE_2_-luciferase and *Renilla* luciferase constructs as in Figure 2B. Wild type BMPRII was co-transfected over a range of concentrations; x-axis displays ng of plasmid. Luciferase assay performed as in Figure 2B. Data represent mean ± SD of a single representative experiment (N = 2 replicates), repeated 3 times (N = 2 replicates) with similar results. *, p≤0.05 compared to 0/siNeg. # p≤0.05 compared to 0/siBMPR2. **B**) Experiment performed as in A, except that tail domain deleted (Δtail) – not wild type – BMPRII was expressed over a range of concentrations. Data represent mean ± SD from one representative of two experiments, each in replicates of N = 2. *, p≤0.05 compared to 0/siNeg.

We noted an interesting phenomenon associated with BMPRII-mediated BRE_2_-luciferase signaling, namely that silencing the receptor promoted this signal, as did its strong overexpression from a CMV-driven promoter (data not shown). We hypothesized that BMPRII may be inhibitory to Smad1 transcriptional activity over a relatively narrow range of expression close to endogenous levels. To examine the effect of BMPRII expression level upon BRE_2_-luciferase signaling, we used siRNA to knock down BMPRII, re-expressed it with increasing amounts of plasmid, and measured BRE_2_-luciferase activity (Figure 5A). The results directly support our hypothesis. Specifically, we found that BMPRII knockdown significantly increases BRE_2_-luciferase activation. With the reintroduction of low amounts of BMPRII, BRE2-luciferase activity is significantly suppressed, and further suppression occurs when a greater amount of BMPRII is reintroduced. However, from the nadir of BRE_2_-luciferase suppression, further increases in BMPRII lead to corresponding increases in BRE_2_-luciferase activity. To evaluate the role of the BMPRII tail domain, the experiment was performed with increasing amounts of Δtail-BMPRII. In contrast to WT BMPRII, Δtail-BMPRII only acts to stimulate BRE_2_-luciferase activation throughout the range of levels examined (Figure 5B). This stimulatory effect is at a very high magnitude compared to that of similar levels of WT-BMPRII. Note the magnitude of the Y axis in Figure 5B (i.e., for Δtail-BMPRII) compared to Figure 5A (WT-BMPRII). These findings demonstrate that BMPRII’s effect on signaling varies in a biphasic fashion as a function of its level of expression. Further, they show that the BMPRII tail domain is a strong suppressor of Smad1 signaling.

### BMPRII Suppresses Signaling of ActRIIA

**Figure 6:**
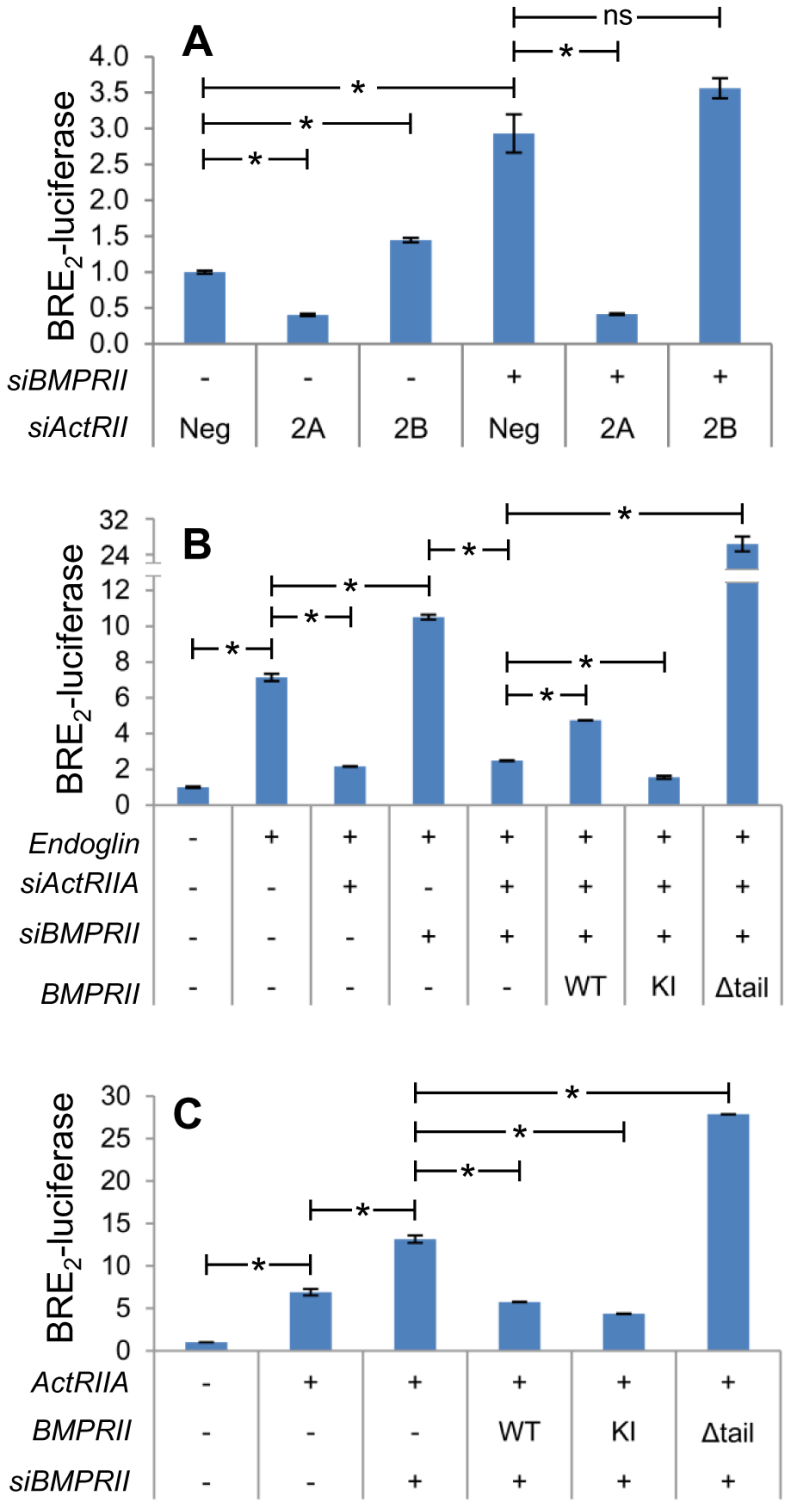
BMPRII suppresses ActRIIA-mediated Smad1 activity. PC3-M cells were transfected with BRE_2_-luciferase, *Renilla* luciferase, and indicated plasmid DNA with or without indicated siRNA. siRNA lanes marked with a hyphen were transfected with non-targeting siRNA. Two days later cells were lysed and luciferase activity was assessed as in Figure 2B. **A)** Increased BRE_2_-luciferase activity upon silencing BMPRII is mediated by ActRIIA. Neg  =  non-targeting siRNA. 2A  =  siActRIIA. 2B  =  siActRIIB. Data represent mean ± SD of a single representative experiment (N = 2 replicates), repeated twice (N = 2 replicates) with similar results. *, p≤0.05 for indicated comparison. **B**) BMPRII-mediated suppression of BRE_2_-luciferase activity is dependent on ActRIIA expression. BMPRII construct abbreviations as in Figure 4. Data represent mean ± SD of a single representative experiment (N = 2 replicates), repeated 3 times (N = 2 replicates) with similar results. *, p≤0.05 for indicated comparison. **C**) BMPRII suppresses signaling from ActRIIA. Data represent mean ± SD from one representative experiment (N = 2 replicates) of three repeated separately (N = 2). *, p<0.05 for indicated comparison.

Increased Smad1 signaling with BMPRII silencing, coupled to the suppressive function of the BMPRII tail domain, led us to hypothesize that BMPRII may suppress Smad1 signaling downstream of another TGFβ superfamily receptor. We therefore examined the extent to which the increased BRE_2_-luciferase signaling seen upon silencing BMPRII is mediated by either ActRIIA or ActRIIB. As before (Figure 2B), silencing of BMPRII increases BRE_2_-luciferase activity (Figure 6A). Importantly, silencing of ActRIIA, but not ActRIIB, significantly suppresses the increased signaling in the face of BMPRII knockdown (Figure 6A, compare column 4, 5, and 6). These findings are consistent with the possibility that BMPRII is suppressing Smad1 signaling by ActRIIA.

We hypothesized that BMPRII-mediated Smad1 suppressive function is dependent on ActRIIA and thus that BMPRII may have altered effects in the absence of ActRIIA. We examined this possibility in endoglin replete cells (Figure 6B). As previously shown, endoglin increases Smad1 signaling, and knockdown of endogenous ActRIIA and BMPRII suppress and increase it, respectively. Also, as before, knockdown of ActRIIA in the face of co-knockdown of BMPRII brings Smad1 signaling back down to the levels observed with ActRIIA suppression alone. Importantly, in the context of concomitant silencing of endogenous ActRIIA and BMPRII, we demonstrate that exogenously restored expression of WT-BMPRII in fact significantly increases Smad1 signaling. This is in contrast to the effect of exogenously restored WT-BMPRII expression in the presence of endogenous ActRIIA (Figure 4C). Importantly, expression of KI-BMPRII in this context has no such effect (i.e. signaling is significantly decreased). This demonstrates that BMPRII’s Smad1 stimulatory activity stems from its kinase function. Finally, expression of Δtail-BMPRII again significantly induces strong signaling, thereby demonstrating the suppressive function of the BMPRII tail domain. These findings demonstrate that in the absence of endogenous ActRIIA, that restoration of BMPRII expression can stimulate BRE_2_-luciferase activity in a kinase-dependent manner.

These findings support the hypothesis that BMPRII may be inhibiting ActRIIA. To test this we exogenously overexpressed ActRIIA and assessed the ability of BMPRII to suppress the resultant increase in BRE_2_-luciferase activity. We found that WT- and KI-BMPRII are able to significantly suppress downstream signaling, while Δtail-BMPRII is not (Figure 6C). Taken together, these results demonstrate that ActRIIA is inhibited by BMPRII, and that this inhibition is dependent upon the BMPRII tail-domain.

### ActRIIA and BMPRII Physically Associate with Endoglin

**Figure 7:**
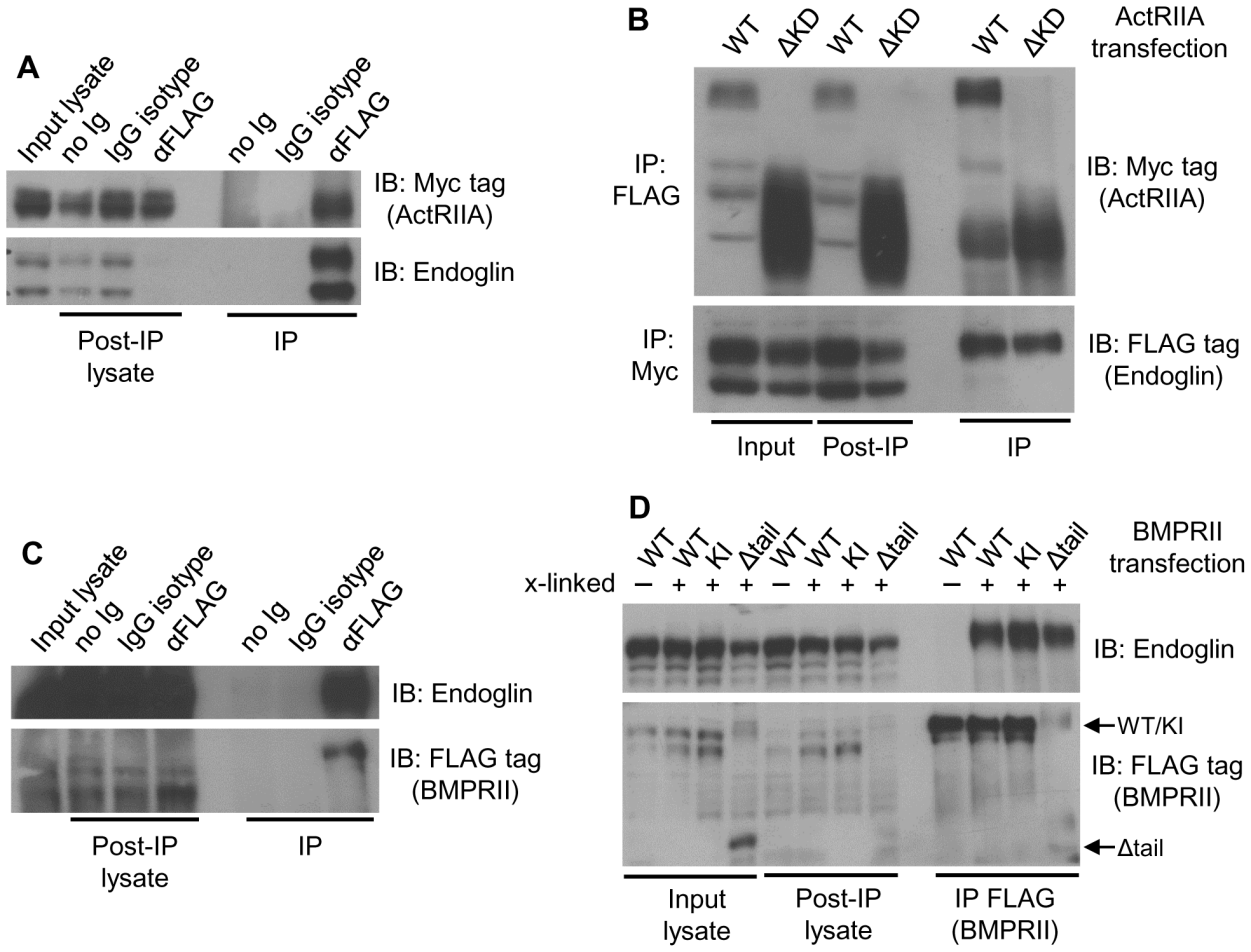
Endoglin physically interacts with ActRIIA and BMPRII. After transient transfection, the surface proteins of PC3-M cells were crosslinked, cells lysed, immunoprecipitation performed, crosslinking reversed, and Western blot performed. **A**) ActRIIA coprecipitates with endoglin. Cells were transfected with Myc-ActRIIA and FLAG-endoglin, FLAG (endoglin) immunoprecipitated, and Western blots probed for ActRIIA (with anti-Myc) and endoglin. Controls for immunoprecipitation included agarose beads alone (no Ig) and nonspecific isotype control IgG (IgG isotype). Input lysate, lysate post-immunoprecipitation (i.e. supernatant), and immunoprecipitation (IP) samples were loaded as indicated. Data are from a representative experiment (N = 2 experiments). **B**) The kinase domain of ActRIIA is dispensable for interaction with endoglin. Cells transfected with Myc-WT or -ΔKD-ActRIIA and FLAG-endoglin as indicated, FLAG or Myc was immunoprecipitated as indicated, and Western blots probed as indicated. Data are from a representative experiment (N = 4 experiments). (**C**) BMPRII precipitates with endoglin. Cells were transfected with FLAG-BMPRII and untagged endoglin, FLAG immunoprecipitated, and Western blots probed as indicated. Data are from a representative experiment (N = 2 experiments). (**D**) The kinase activity and tail domain of BMPRII are dispensable for interaction with endoglin. Cells transfected with FLAG-WT, -KI, or -Δtail-BMPRII and untagged endoglin as indicated, FLAG-BMPRII immunoprecipitated, and Western blots probed as indicated. In some instances surface proteins were crosslinked (+), which was reversed after immunoprecipitation, while in other instances proteins were not crosslinked (-) Data are from a representative experiment (N = 3 experiments).

The functional interaction between endoglin, ActRIIA and BMPRII supports the notion that they physically interact. Endoglin and ActRIIA have been shown to physically interact in monkey fibroblast COS1 cells [44]. To assess whether physical interactions were occurring in human prostate epithelial cells, cells were transfected with Myc-tagged ActRIIA and FLAG-tagged endoglin, cell surface proteins crosslinked, and endoglin immunoprecipitated from lysates with anti-FLAG antibody. Crosslinking was then reversed and immunoprecipitates probed for endoglin and ActRIIA by Western blot (Figure 7A). In this manner it is shown that ActRIIA is detected after endoglin immunoprecipitation, is not detected in isotype antibody or no antibody controls, and that endoglin and ActRIIA are present in lysates and that there is efficient endoglin immunoprecipitation. To assess whether the kinase domain of ActRIIA is required for interaction, cells were transfected as above using either WT or ΔKD-ActRIIA, and immunoprecipitation performed of either endoglin (FLAG) or ActRIIA (Myc; Figure 7B). An endoglin/ActRIIA complex is demonstrated by reciprocal co-immunoprecipitation, and the kinase domain is shown not to be necessary for the interaction.

To determine whether BMPRII interacts with endoglin, cells were transfected with FLAG-BMPRII and untagged endoglin, crosslinked, and FLAG-BMPRII immunoprecipitated (Figure 7C). BMPRII is detected after endoglin immunoprecipitation, is not detected in controls, and Western blotting demonstrates that high levels of endoglin expression are achieved in lysates, while levels of BMPRII are much lower. To determine whether the kinase activity or tail domain of BMPRII is required for interaction, cells were transfected with FLAG-tagged WT, KI, or Δtail-BMPRII and untagged endoglin (Figure 7D). In addition, we assessed whether crosslinking was required to reveal such an interaction. We find that the kinase activity and tail domain of BMPRII are not required for interaction with endoglin, but that the complex cannot be detected without crosslinking cell surface proteins. These findings suggest that the interaction is weak, present at low levels, and/or is lost upon cell lysis. Taken together, the results demonstrate that endoglin physically interacts with both ActRIIA and BMPRII, that the kinase activity of neither receptor is required, that the tail domain of BMPRII is not required, and that the interaction likely involves the extracellular regions of the receptors.

### ActRIIA and BMPRII Form a Complex in Prostate Cancer Cells

**Figure 8:**
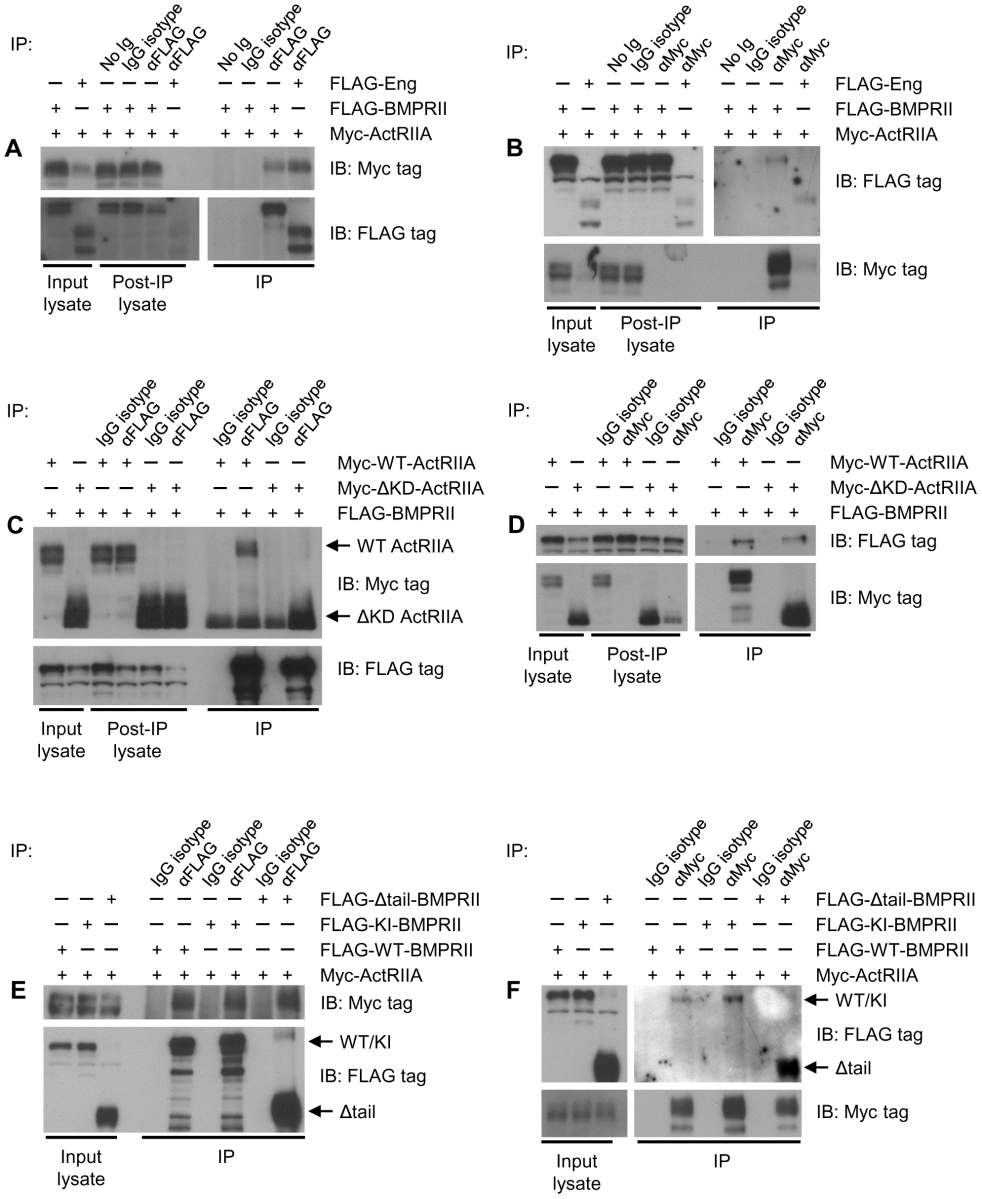
ActRIIA and BMPRII physically interact . Coimmunoprecipitation experiments were performed as in Figure 7. In all experiments, cells were transfected, cell surface proteins crosslinked, immunoprecipitation (IP) performed, crosslinks reversed, and Western blot (IB) performed as indicated. In some studies, cells were transfected with FLAG-endoglin as a positive control. Input lysate, post IP lysate, and IP samples are loaded as indicated. (**A and B**) ActRIIA precipitates with BMPRII. (**C and D**) ActRIIA kinase domain is dispensable for interaction with BMPRII. (**E and F**) BMPRII kinase activity and tail domain are dispensable for interaction with BMPRII. All data are from a representative experiment, repeated at least N = 5 separate times.

Work presented above demonstrates that ActRIIA and BMPRII functionally interact in PCa cells. Moreover, each RII forms a complex with endoglin. In order to assess whether ActRIIA and BMPRII are physically present in the same complex, cells were transfected with Myc-ActRIIA and FLAG-BMPRII, cell surface proteins were crosslinked, and complexes immunoprecipitated, crosslinking reversed, and Western blot performed, as above. As a positive control, cells were also transfected as indicated with endoglin, and endoglin’s physical interaction with each RII was assessed simultaneously. In all other conditions, cells were not transfected with endoglin. We have previously demonstrated that the level of endoglin protein expression in these cells is below the level of detection [45]. This therefore allows us to assess ActRIIA and BMPRII interactions in the face of undetectable levels of endoglin protein. As can be seen in Figure 8A, ActRIIA is detected upon immunoprecipitation of BMPRII, but not upon immunoprecipitation with isotype control IgG or beads alone. Further, ActRIIA and BMPRII or endoglin are expressed in lysates, and immunoprecipitation was efficient. Note that in Figures 8A and 8B, less input protein was used to assess the endoglin-RII interaction than that of ActRIIA and BMPRII. In the reciprocal situation, BMPRII is only detected upon immunoprecipitation with ActRIIA, and not with nonspecific controls (Figure 8B).

To determine whether the kinase domain of ActRIIA is required for the interaction, cells were transfected with FLAG-BMPRII and with Myc-WT ActRIIA or Myc-ΔKD-ActRIIA, and FLAG-BMPRII immunoprecipitated. Both WT ActRIIA and ΔKD-ActRIIA constructs can be found in precipitates of WT BMPRII (Figure 8C). Note that because of the similarity in size between ΔKD-ActRIIA and the IgG heavy chain, complete separation of the signal could not be obtained. Thus, with immunoprecipitation, an IgG signal is observed in all lanes, while in the ΔKD-ActRIIA lane a much stronger and broader signal is observed. Similarly, WT BMPRII can be found in precipitates of either WT or ΔKD-ActRIIA (Figure 8D). These findings demonstrate that ActRIIA interacts with BMPRII independent of the ActRIIA kinase domain, and that such interaction can occur in the absence of endoglin.

To assess whether the kinase activity or tail domain of BMPRII is required for BMPRII and ActRIIA interaction, experiments were performed in which either WT, KI, or Δtail-BMPRII was immunoprecipitated, and associated WT ActRIIA detected by Western blot. WT ActRIIA is found in precipitates of all three BMPRII constructs (Figure 8E). Reciprocally, immunoprecipitation of ActRIIA demonstrates bound WT, KI, or Δtail-BMPRII (Figure 8F). Taken together, these findings demonstrate that ActRIIA and BMPRII form a physical complex independent of ActRIIA’s kinase domain and BMPRII’s kinase activity and tail domain. Further, such interactions occur in the absence of detectable levels of endoglin, indicating that it is not necessary. Like the RII complexes with endoglin, this suggests that the extracellular domains are likely mediating the interaction. However, because the transmembrane domain and a shortened portion of the intracellular domain remains in the truncated receptors, their involvement in mediating receptor interactions cannot be ruled out at this time.

### Endoglin-Mediated Suppression of Invasion is Dependent on ActRIIA Kinase Domain and Independent of BMPRII Kinase Activity or Tail Domain

**Figure 9:**
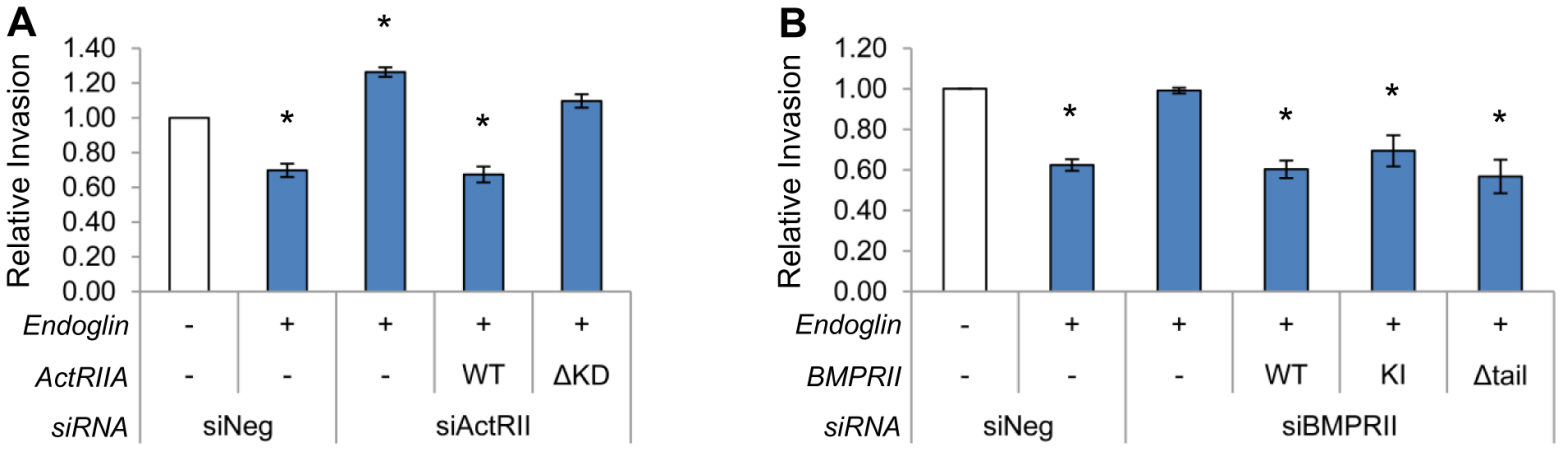
Endoglin-Mediated Suppression of Invasion is Dependent on ActRIIA Kinase Domain and Independent of BMPRII Kinase Activity or Tail Domain. Cells were transfected with endoglin, ActRIIA (A) or BMPRII constructs (B), and ActRIIA or BMPRII directed siRNA, as indicated, and cell invasion assays conducted as in Figure 1. Data represent the mean ± SEM of N = 2 independent experiments (A) or N = 3 independent experiments (B), each in replicates of 3. * denotes p≤0.05 compared to cells transfected with empty vector and non-targeting siRNA.

To further evaluate RII function in regulating invasion, we assessed the ability of mutant RIIs to restore endoglin mediated suppression of invasion (EMSI) in cells expressing endoglin but lacking ActRIIA or BMPRII (Figure 9). In this set of experiments, cells were transfected with endoglin, demonstrating EMSI. Upon knockdown of either ActRIIA (Figure 9A) or BMPRII (Figure 9B), EMSI was reversed. Restoration of WT- but not ΔKD-ActRIIA expression rescues EMSI in cells where endoglin was expressed and endogenous ActRIIA was knocked down. For BMPRII, restoration of WT, KI, or Δtail-BMPRII restores EMSI. Thus, ActRIIA promotes EMSI via its kinase domain, while BMPRII acts independent of its kinase activity or tail domain.

## Discussion

Endoglin has previously been shown to be an important suppressor of human PCa cell invasion and metastasis, and its expression is lost with cancer progression [14], [31]–[33]. Further, it has been shown to serve a gatekeeper function in regulating signaling through the TGFβ superfamily of receptors in several cell types, including prostate [14], [23], [24]. It is therefore important to increase our understanding of endoglin function. In this study we demonstrate for the first time that two distinct RIIs – ActRIIA and BMPRII – are required for endoglin-mediated suppression of invasion (EMSI). This was demonstrated through approaches involving changing the level of receptor expression, as well as through approaches involving disruption of ligand binding. The latter approach utilized cells with endogenous RII expression, thereby supporting the true physiological relevance of our findings. Also, our findings are supported by studies conducted by others in humans, and in fact suggest a mechanistic explanation for them. Specifically, loss of BMPRII expression has been associated with more advanced tumors and poorer survival in patients with PCa [46], [47]. Our studies demonstrate that loss of BMPRII precludes endoglin from suppressing invasion, which in humans would be predicted to translate into poorer survival due to the increased development of metastatic disease.

Interestingly, we found that ActRIIA and BMPRII have opposing actions on Smad1, a key downstream effector of the pathway previously shown to be necessary for EMSI. We show that ActRIIA promotes Smad signaling predominantly through Smad1, with a minor role for Smad5 and none for Smad8. Further, we demonstrate that the ActRIIA kinase domain is necessary for Smad1 activation.

Ultimately, BMPRII can be considered to have a biphasic role in regulating Smad1 activation. This is dependent upon several factors, including the level of receptor expression as well as upon the presence of ActRIIA. Low levels of endogenous BMPRII expression suppress Smad1 signaling, an effect appreciated upon BMPRII knockdown. This is independent of its kinase function, but is dependent on its tail domain. As BMPRII expression is exogenously raised beyond a threshold, it increasingly activates Smad1 signaling. By contrast, in the absence of endogenous ActRIIA, even modest expression of BMPRII promotes Smad1 activity, and this is dependent upon BMPRII’s kinase domain. We thus propose that BMPRII suppresses ActRIIA-mediated Smad1 signaling, and that this is mediated in a BMPRII tail domain-dependent manner. At endogenous levels of expression, this effect predominates. As BMPRII expression increases, it surpasses the amount required to mediate the tail-domain-dependent suppressive effects on endogenous ActRIIA, and the kinase-dependent promotion of Smad1 signaling becomes predominant. The biphasic action of BMPRII may explain why many reports in the literature indicate that BMPRII stimulates Smad1 activation, while others indicate the opposite [48], [49].

The functional interaction between endoglin, ActRIIA, and BMPRII led us to demonstrate for the first time that they all physically interact. First, we demonstrate that endoglin interacts with both ActRIIA and BMPRII. This occurs independent of the kinase domain of ActRIIA, and independent of the kinase activity and tail domain of BMPRII. This suggests that the interaction likely occurs minimally through extracellular domains. We cannot exclude the possibility that the cytoplasmic domains of these receptors contribute to interactions. In fact, it has previously been shown that interactions between endoglin and TβRII, ALK5, and ALK1 involve interactions between extracellular domains, as well as interactions between cytoplasmic domains [12], [50]. While an endoglin interaction with ActRIIA has been previously observed [44], these authors failed to find an interaction with BMPRII. This work was largely performed in COS1 cells, derived from the kidney of the African green monkey. The differences between these studies likely reflect differences in the complement of additional regulatory factors between the cells examined. Finally, it is important to consider that our data does not differentiate between endoglin interacting with a large complex containing both ActRIIA and BMPRII or with separate pools containing each ActRIIA and BMPRII individually.

We report for the first time, to our knowledge, a physical interaction between ActRIIA and BMPRII. Like the interaction between endoglin and these RIIs, this is also independent of ActRIIA’s kinase domain and BMPRII’s kinase function and tail domain. These findings shed light on a previous report of monocyte chemotaxis which demonstrated functional cooperativity between ActRIIA and BMPRII in response to BMP7, which led the authors to propose complexes containing both ActRIIA and BMPRII [51]. Our data demonstrate the existence of such complexes – potentially heterodimers – in PCa cells. We propose that it is in these complexes that the BMPRII tail domain suppresses the Smad1 signaling function of ActRIIA.

It will be important for future investigations to determine the mechanism by which BMPRII suppresses ActRIIA-mediated Smad1 signaling. In this regard it should be noted that the long cytoplasmic tail of BMPRII is a unique feature among the RIIs, and its role as a scaffold and modulator of various signaling proteins is increasingly being appreciated [39], [52]. Further, several studies, when considered together, serve to frame a functional role for BMPRII as a modulator of ActRIIA. Specifically, in a series of murine-based studies, loss of BMPRII increases ActRIIA-mediated BMP6/7 signaling in pulmonary artery smooth muscle cells [49]. The role of the tail domain in this context was not explored. It is instructive, however, that similar phenotypes (pulmonary hypertension) in mouse models are observed upon (1) BMPRII tail domain truncation or (2) dominant negative expression in smooth muscle, or upon (3) germline heterozygous deletion [53]–[55]. Moreover, mutations in *BMPR2* are associated with familial and sporadic primary pulmonary hypertension (PPH) in humans [56]–[58], and it is notable that many PPH-causing mutations result in truncation of the tail domain [59], [60]. Together, these studies suggest that mechanisms operating through the tail domain play an integral role in BMPRII’s biological functions and highlight the importance of understanding them, including the regulation of ActRIIA signaling.

In considering the mechanism(s) by which BMPRII suppresses ActRIIA-mediated Smad1 signaling in the current system, there are several non-mutually exclusive possibilities. A probable mechanism is that the BMPRII tail domain mediates signaling via noncanonical accessory proteins, many of which interact via the tail domain [61]–[63]. A second possible mechanism is that the BMPRII tail domain interacts directly with ActRIIA, as could occur in the ActRIIA/BMPRII complexes we have identified. If this were the only mechanism operative in our system, it would require that the BMPRII tail domain have dual and opposing functions. One would be a Smad1 activating function, presumably through the ability of the tail domain to facilitate the ability of ActRIIA to form a functional signaling receptor complex with endoglin-ALK2. The other function would inhibit Smad1 activation, presumably through sequestration of ActRIIA. While these are complex requirements, it should be noted that the tail domain is in fact large and appears to possess several biological functions. Ongoing investigations in our group are seeking to elucidate the mechanism.

We show that endoglin suppresses invasion dependent on the kinase domain of ActRIIA. Considered with our previous work [14], this suggests the existence of a complex of receptors containing endoglin, ALK2, and ActRIIA that signal through Smad1 to suppress PCa invasion. This provides for a complex containing both a RI and a RII (i.e., ALK2 and ActRIIA, respectively), which are essential for canonical signaling through the TGFβ superfamily of receptors. BMPRII is also required for EMSI, but independent of its kinase activity or tail domain. Notably, then, the ability of BMPRII to mediate EMSI does not correlate with its regulation of Smad1 signaling. This suggests that there may be a second, Smad-independent pathway promoted by BMPRII that is simultaneously required for EMSI.

Areas of overlap between endoglin and BMPRII biology may shed light on future investigations designed to further understand their co-regulation of motility. First, both endoglin and BMPRII interact with a series of proteins with LIM domains. Specifically, endoglin’s interactions with zyxin [15] and zyxin-related protein 1 [16] regulate the composition of focal adhesions and the cytoskeleton. BMPRII interacts with LIMK1 to regulate dendrite outgrowth [39], [64] and with FHL2 to regulate chromatin remodeling and thus expression of a subset of target genes [65]. Notably, however, these interactions with BMPRII occur via the cytoplasmic and tail domain. If they are responsible, it may be that endoglin recruits them to an endoglin/BMPRII complex, while BMPRII is required for their relevant activation. A second area of overlap involves interaction with members of the dynein family of motor proteins. In particular, endoglin and BMPRII have been shown to interact with Tctex2β and Tctex1, respectively [63], [66], and both participate in the regulation of cell motility, primarily through effects upon microtubules. Given the size of BMPRII and the complexity of it biology, particularly in the tail domain, there are numerous additional proteins that interact with BMPRII (reviewed in [67]) which might also contribute. Future investigations will aim to further characterize the role of these other proteins in BMPRII’s regulation of prostate cell motility.

Our data using ligand traps suggests that EMSI is a ligand-mediated event. There are more than 20 known BMP family members. These can be divided into structurally and functionally related classes, including those for BMP2/4, BMP5/6/7, and BMP9/10 [68]. Given the receptors identified in this study, several reports in the literature suggest ligands that may be operative in this system. BMP9/10 signals through endoglin, ActRIIA, and BMPRII to inhibit migration in endothelial cells [69]. Loss of BMPRII in pulmonary artery smooth muscle cells augments signaling of BMP6/7 though ActRIIA, while BMP2/4 signaling is dampened [49]. This group has also shown that BMPRII and ActRIIA differentially regulate signaling and function downstream of BMP4 and BMP7, and that this effect is dependent on the level of receptor expression [70]. Moreover BMP ligands regulate diverse facets of PCa biology, with some studies find tumor promoting effects [71]–[75], while others suggest inhibitory roles [76]–[78]. Of note, BMPRII is frequently lost from prostate cancer epithelium [46], [47], as is BMP7 [47]. This loss correlates with advanced grade and stage, and poorer survival [46]. Dominant negative BMPRII expression in autochthonous mammary cancer mouse model promotes development of metastasis [79], and loss of BMP10 correlates with disease progression in human patients [80]. In the current study we demonstrate that Smad1 phosphorylation downstream of BMP7 and BMP9 is regulated by ActRIIA and BMPRII in a manner consistent with our other findings in this study. Taken together, our current findings, along with prior studies by us and others, suggest that the BMP5/6/7 and/or BMP9/10 subfamilies may be responsible for the effects observed in this study.

**Figure 10:**
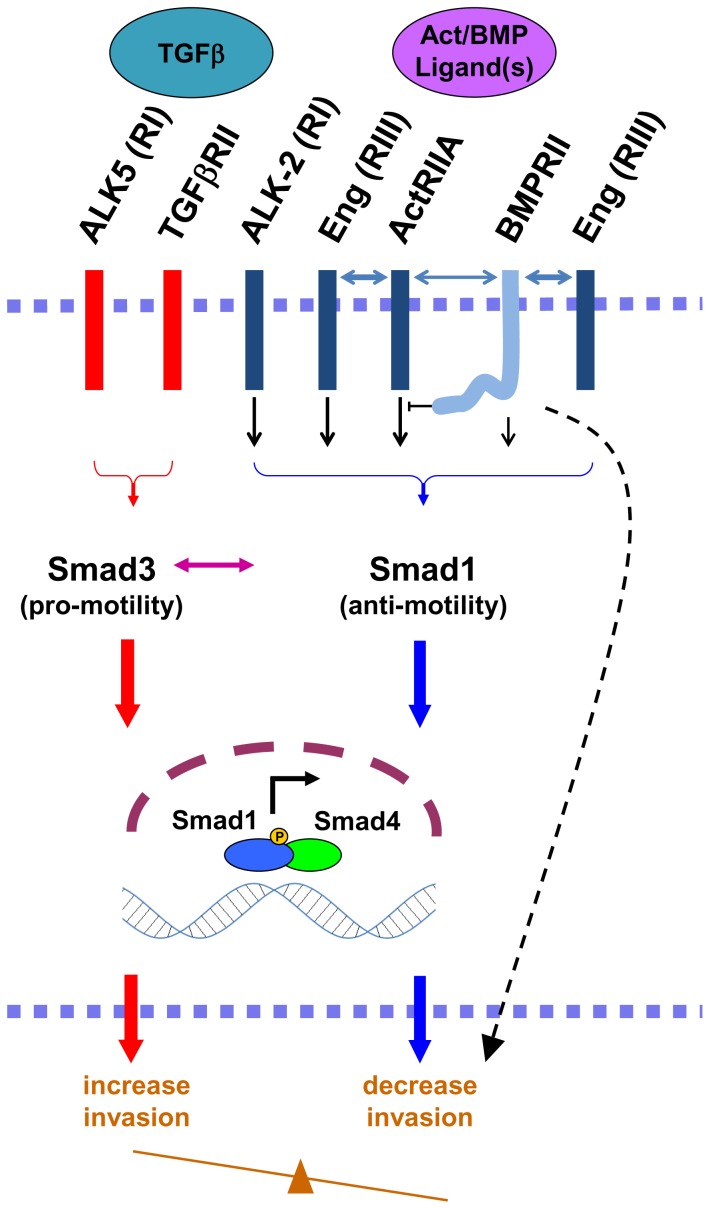
Proposed model for the regulation of endoglin-mediated suppression of invasion by ActRIIA and BMPRII. Based upon our current and prior findings, we propose the model depicted in this schema. A ligand-stimulated endoglin-ActRIIA-ALK2 signaling axis promotes Smad1 signaling to decrease the invasiveness of PCa cells. BMPRII is simultaneously required through additional, noncanonical regulatory elements (depicted as a dashed arrow). See text for expanded discussion. BMPRII plays a bimodal role in Smad1 signaling, promoting it via the kinase domain while inhibiting it in a tail-domain-dependent manner, potentially through a tail-domain-interacting protein or by direct interaction with ActRIIA. Endoglin physically interacts with both ActRIIA and BMPRII, and BMPRII interacts with ActRIIA in the absence of endoglin (bidirectional arrows). Previous work from our group has demonstrated that TGFβ signals through Smad3 to promote PCa invasion, that the balance between Smad3 and Smad1 regulates motility and invasion, and that endoglin acts as a gatekeeper in this regard.

The current findings, coupled with our prior ones [14], [31]–[33], [81], [82], support the model proposed in Figure 10. We propose an ActRIIA-ALK2-Smad1 axis promoted by endoglin which functions to suppress PCa invasion. BMPRII is also necessary for EMSI. Additional regulatory factors appear to be necessary. This is supported by prior findings that Smad1 activation is known to be sufficient to suppress invasion in the absence of endoglin [14], coupled to current findings that there is loss of EMSI upon silencing of BMPRII despite the resultant increase in Smad1 activation. That the tail domain is required for Smad1 suppression but not for EMSI demonstrates that these are uncoupled processes. Whether this is due to an alternative signaling pathway, the interaction of BMPRII with ActRIIA, or the combination of both has yet to be determined. Together, the current findings provide new information about endoglin pathway signaling in human PCa. We have previously described endoglin’s ability to act as gatekeeper in regulating human PCa cell signaling and invasion, acting to stimulate the Smad1 anti-motility pathway, thereby decreasing the relative contribution of the Smad3 pro-motility pathway [14]. In the pro-motility pathway, the TGFβ ligand binds to the RII-RI receptor complex composed of TGFβRII-ALK5, activates Smad3, and thereby stimulates invasion. The relative activation of the Smad1 and Smad3 pathways determines the invasive capacity of the cells.

In summary, we have identified the two type II TGFβ superfamily receptors, ActRIIA and BMPRII, as necessary for endoglin-mediated suppression of invasion in human PCa cells. These have opposing effects on the required downstream effector Smad1. ActRIIA signals through its kinase domain through Smad1 to suppress invasion. BMPRII acts independent of its regulation of Smad1. We also show that BMPRII-mediated antagonism of ActRIIA is dependent on the BMPRII tail domain and independent of its kinase function. We demonstrate that BMPRII has biphasic signaling capabilities, dependent upon level of expression and the presence of ActRIIA. We confirm physical interaction of ActRIIA with endoglin, demonstrate a physical interaction between BMPRII and endoglin, and identify a novel interaction between ActRIIA and BMPRII. Together, these findings shed new light on how this family of receptors collectively cooperates to regulate cell signaling and function central to PCa progression.

## Supporting Information

Figure S1 **Restoring RII expression to near-endogenous levels.** Cells were simultaneously transfected with 40 nM siRNA (either non-targeting [N] or targeting ActRII or BMPRII) and increasing amounts of plasmid DNA (expressed in ng/ml). Panels depict mRNA expression of cells expressing WT ActRII (A), ΔKD ActRII (B), WT BMPRII (C), or Δtail BMPRII (D). Data represent mean ± SD from a single experiment conducted in replicates of N = 2, that was repeated 3 separate times (also in replicates of N = 2) with similar results. Conditions used for KI BMPRII, which was established by point mutation of the WT construct, were identical to those for WT BMPRII.
(TIF)
