# Supplementary figures and images for: Endoglin-Mediated Suppression of Prostate Cancer Invasion Is Regulated by Activin and Bone Morphogenetic Protein Type II Receptors
Source: PLoS One. 2013 Aug 13;8(8):e72407. doi: 10.1371/journal.pone.0072407 (PMC3742533; doi:10.1371/journal.pone.0072407)

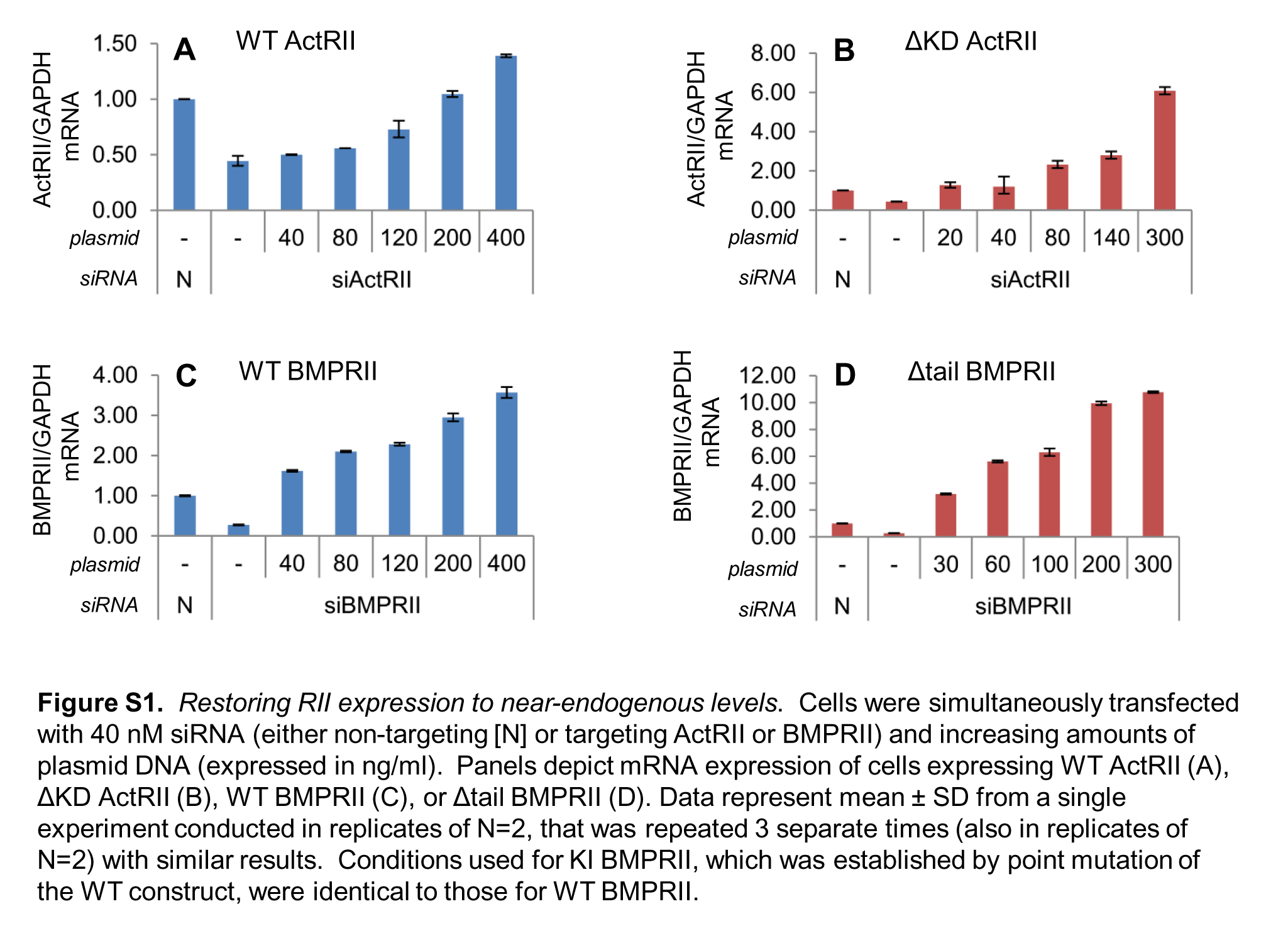

Supplement: Figure S1 — Restoring RII expression to near-endogenous levels. Cells were simultaneously transfected with 40 nM siRNA (either non-targeting [N] or targeting ActRII or BMPRII) and increasing amounts of plasmid DNA (expressed in ng/ml). Panels depict mRNA expression of cells expressing WT ActRII (A), ΔKD ActRII (B), WT BMPRII (C), or Δtail BMPRII (D). Data represent mean ± SD from a single experiment conducted in replicates of N = 2, that was repeated 3 separate times (also in replicates of N = 2) with similar results. Conditions used for KI BMPRII, which was established by point mutation of the WT construct, were identical to those for WT BMPRII. (TIF) [file pone.0072407.s001.tif]
